# Analysis of factors associated with ovarian reserve in a group of poor responders to in vitro fertilization: A cross-sectional study

**DOI:** 10.18502/ijrm.v18i12.8028

**Published:** 2020-12-21

**Authors:** Budi Wiweko, Quamila Fahrizani Afdi, Achmad Kemal Harzif, Gita Pratama, Kanadi Sumapradja, Raden Muharam, Andon Hestiantoro, Sarah Chairani Zakirah

**Affiliations:** ^1^Department of Obstetrics and Gynecology, Division of Reproductive Endocrinology and Infertility, Faculty of Medicine, Universitas Indonesia, Jakarta, Indonesia.; ^2^Yasmin IVF Clinic, Dr. Cipto Mangunkusumo General Hospital, Jakarta, Indonesia.; ^3^Human Reproductive, Infertility and Family Planning Research Center, Indonesia Medical Education and Research Institute (IMERI), Faculty of Medicine, Universitas Indonesia, Jakarta, Indonesia.

**Keywords:** Ovarian reserve, Poseidon, In vitro fertilization.

## Abstract

**Background:**

Poor ovarian reserve and a high rate of pregnancy failure associated with low quality and quantity of oocytes are observed in poor responders to in vitro fertilization.

**Objective:**

To assess the effect of age, body mass index (BMI), endometriosis, and history of ovarian surgery on ovarian reserve in a group of poor responders.

**Materials and Methods:**

In this cross-sectional study 749 women who referred to Yasmin Clinic of Dr. Cipto Mangunkusumo National General Hospital from January 2013 to June 2017 were enrolled. Two definitions of poor responders and Poseidon criteria and consecutive sampling techniques were used. Participants were divided into good and poor responder groups based on the ovarian reserve test; participant with oocyte ≤ 3 was classified as a poor responder. Based on this, 188 participants with nine (4-47) oocytes were included in the poor responder group. While, good responder comprised of two (0-3) oocytes.

**Results:**

Age and anti-Mullerian hormone level (AMH) were significantly associated with ovarian reserve in the poor-responder group (p < 0.001). However, in multivariate analyses, age was the only significant predictor of ovarian response in the poor-responder group (p = 0.004). While endometriosis was the significant predictor of Poseidon groups 1 and 4, surgical history was the significant predictor of Poseidon groups 2 and 3. Meanwhile, an increase in BMI decreased the risk of classification under Poseidon group 3.

**Conclusion:**

Age, AMH, BMI, endometriosis, and history of ovarian surgery affected the risk of classification of the Poseidon group.

## 1. Introduction

Infertility are generally classified into good and poor responder. Assisted reproductive technology (ART) is a medical procedure used to treat infertility by directly manipulating oocytes outside the body. One of the most common types of ART is in vitro fertilization (IVF) (1). Approximately 9-24% of women are poor responders, and this number has increased in the last decade (2). Some study have shown that poor responders are at risk of developing pregnancy complications, such as hypertension and preeclampsia, due to low-quality embryo (3, 4). About 56% of sub fertile couples worldwide undergo IVF, with a success rate of 30-40% (5). In 2011, 2,627 IVF cases from 14 IVF clinics were recorded in Indonesia, with a success rate of 38.5% (6). According to the Yasmin Clinic Dr. Cipto Mangunkusumo National General Hospital (RSCM), 318 IVF cycles were recorded in 2013 and 551 cycles in 2014. Participants as much as 39 women were recorded as poor responders in 2013 and 24 women in 2014 (7, 8).

Response to ovarian stimulation by examining ovarian reserve is important to determine the success of ART (9). The poor-responder group is a group of participants who have ≤ 3 oocytes after ovarian stimulation (10). In 2011, the Bologna criteria indicated that a poor responder must meet two of the three criteria, which include age > 40 yr or presence of risk factors, antral follicle count (AFC) < 6-8 follicles per ovary or anti-Müllerian hormone (AMH) level < 0.5-1.1 ng/mL, and a history of producing ≤ 3 oocytes after ovarian stimulation (2). However, Poseidon classification is the current criteria recommended by the European Society of Human Reproduction and Embryology (ESHRE) guideline. Ovarian reserve marked by low quantity of oocytes is associated with the quality of IVF outcomes. Ovarian reserve refers to the number, size, and quality of primordial follicles in the ovary and the reproductive potential of each oocyte (11). The best parameters to identify ovarian reserve conditions are age, AMH level, and AFC (12). An association was observed between AMH level and the number of follicles in the ovary. A study in Indonesia has shown that an AMH level of 1.40 µg/mL and AFC at 7 is the cut-off values used to predict good response and ovarian condition (13).

Ovarian reserve is typically affected by various factors such as age, body mass index (BMI), endometriosis, history of ovarian surgery, chemotherapy, and radiotherapy (14-16). Participants at 35 yr of age affect the low number and quality of primordial follicles, thereby affecting ovarian reserve and infertility (14). In addition, BMI also affects the ovarian reserves by changing the metabolism and hormone level of women (17). Moreover, a history of ovarian surgery can reduce ovarian reserve due to vascular injury, infection after surgery, and micro thromboembolism (14). Endometriosis is the other factor affecting ovarian reserves particularly in young participants (18). The association between decreased ovarian reserve and endometriosis caused by chronic inflammation that affects dormant follicles in the ovarian cortex has also been reported (19).

This study aimed to determine the most significant factors affecting poor ovarian response and the severity of the effect on decreased ovarian reserve based on the Poseidon group classifications. The first objective of this study was to find the most significant association between the six independent variables- that is age, BMI, endometriosis, endometriosis location, history of ovarian surgery, and AMH- and poor-responder participants women. The second goal was to find some association between each Poseidon group and three independent variables as the factors, that is BMI, history of surgery, and endometriosis.

## 2. Materials and Methods

This cross-sectional study was conducted from January 2013 to June 2017. Data were obtained by consecutive sampling. Participants data, such as age, BMI, endometriosis, and history of ovarian surgery, were obtained from medical records. Women who had undergone controlled ovarian stimulation protocol of IVF using gonadotropin, those who were assessed for ovarian reserve based on the number of oocytes via ovum pick-up, and those classified as good and poor responders were included. However, women with infertility due to congenital or anatomical abnormality were excluded from the study.

In this study, participants were divided into good and poor responder groups based on the ovarian reserve test; participant with oocyte ≤ 3 was classified as a poor responder. Based on this, 188 participants with nine (4-47) oocytes were included in the poor responder group. While, good responder comprised of two (0-3) oocytes. Then, based on the Poseidon criteria- which is the criteria of poor responder risk factor, the poor-responder participants were classified. The Poseidon criteria comprised into four groups, as shown in Table I.

### Antagonist protocol

Gonadotropin injection was started on day 2 or 3 of the period with initial doses between 300 and 375 IU. Meanwhile, GnRH antagonist was injected 0.25 mg daily starting on day 6 of the stimulation or when the diameter of the leading follicle was 14 mm. Ovulation trigger with rhCG 250 ug was initiated when at least three follicles reached a diameter of ≥ 17 mm. Ovum pick-up was done approximately 36 hr after oocyte maturation which was triggered with rhCG.

**Table 1:** Classification of Poseidon


**Poseidon group**	**Age (yr)**	**AFC (follicle)**	**AMH (ng/ml)**	**The number of oocytes in the previous stimulation**
**Group 1**	< 35	≥ 5	1.2	Subgroup 1a: <4 oocytes Subgroup 1b: 4-9 oocytes
**Group 2**	≥ 35	≥ 5	1.2	Subgroup 2a: <4 oocytes Subgroup 2b: 4-9 oocytes
**Group 3**	< 35	< 5	< 1.2	—
**Group 4**	≥ 35	< 5	< 1.2	—
Ovarian responses are classified into four groups. Poseidon group 1 and 3 are group of women aged < 35 years, while Poseidon group 2 and 4 are the group of women aged ≥ 35 years. Adopted from Alviggi C *et al*, Fertil Steril, 2016 (20), AMH: Anti-mullerian hormone; AFC: Antral follicle count				

### Ethical consideration

This study was approved by the ethics committee of the Faculty of Medicine on August 06, 2018 (reference number: 0781/UN2.F1/ETHIC/2018). Informed consent was obtained from all participants before study enrollment.

### Statistical analysis 

Data were analyzed via bivariate tests using Chi-square test and Mann-Whitney U-test, and a multivariate analysis using linear regression was conducted to assess factors that lead most to the low ovarian reserve in poor-responder participants. A p-value < 0.05 was considered as statistically significant. Confidence interval 95% (CI 95%) was the benchmark to evaluate the data. Data were analyzed using the Statistical Package for the Social Sciences software version 20.0, SPSS Inc, Chicago, Illinois, USA (SPSS).

## 3. Results

### Characteristics of the participants

A total of 749 participants underwent ovarian stimulation and met the eligibility criteria from 2013 to 2017. Based on the Poseidon criteria, 262 (35%) participants were included in the non-Poseidon group, seven (0.9%) in the Poseidon 1a group, 64 (8.5%) in the Poseidon 1b, 30 (4%) in the Poseidon 2a, 113 (15.1%) in the Poseidon 2b, 73 (9.8%) in the Poseidon 3, and 200 (26.7%) in the Poseidon group 4.

Table II presents the characteristics of participants based on ovarian reserve test. Based on the number of oocytes, 188 (25%) participants were included in the poor-responder and 561 (75%) in the good-responder groups. The age of the poor-responder participants was 38.5 (25-50) years, primary infertility was reported in 170 (90.4%) respondents, the average durations of infertility was 8 yr (range: 1-23), the average BMI was 23.37 kg/m${}^{2}$, the average number of oocytes was 2 (0-2), and the average AMH levels were 0.55 (0.01-4.65). In this study, a majority of the, that is, 135 (71.8%) participants, did not have endometriosis. The main location of endometriosis was in the ovarium in 30 (56.6%) participants. Most participants in both groups did not have a history of ovarian surgery - 164 (87.2%) participants in the poor-responder group.

### Relationship between some factors and poor ovarian response according to oocyte count

The association between age, BMI, endometriosis, and history of ovarian surgery with poor-responder group are presented in Table III.

It is also shows that age ≥ 35 yr was a significantly strong risk factor in poor-responder group. Furthermore, endometriosis was twice as a higher risk factor for them. However, no association was observed between the location of endometriosis and the poor responders (p = 0.105). A history of ovarian surgery was significantly 2.3 times higher a risk factor for poor responders. However, further analysis using logistic regression, which included AMH as an additional cofounding factor, showed that only age (p = 0.004) and AMH level (p < 0.001) were significantly associated with the poor-responder group. Meanwhile, BMI, endometriosis, and history of ovarian surgery were not significantly associated with the incidence of poor-response. Table IV shows the multivariate analysis of each Poseidon group. Endometriosis was a significant predictor of classification under Poseidon groups 1 and 4 - with an odds ratio of 2.5 and 2.7, respectively. A history of ovarian surgery was a significant determinant of inclusion in Poseidon groups 2 and 3 (p < 0.05), whereas an increase in BMI decreased the risk of inclusion in Poseidon group 3.

**Table 2:** Characteristics of participants


**Demographical and clinical characteristics**		**Good-responder group (n = 561)**	**Poor-responder group (n = 188)**
Age (yr)*		35 (22-48)	38.5 (25-50)
Infertility **			
	**Primary**	302 (53.8)	170 (90.4)
	**Secondary**	259 (46.2)	41 (21.8)
Length of infertility (yr)*		7 (1-23)	8 (1-23)
BMI (kg/m${}^{2}$)*		23.63 (15.63-43.28)	23.37 (15.43-38.87)
Number of oocytes*		9 (4-47)	2 (0-3)
AMH*		2.68 (0.01-34.06)	0.55 (0.01-4.65)
Endometriosis**			
	**Yes**	92 (16.4)	53 (28.2)
	**No**	469 (83.6)	135 (71.8)
Location of endometriosis**			
	**Ovarium**	67 (72.8)	30 (56.6)
	**Extra ovarium**	10 (10.9)	7 (13.2)
	**Both**	15 (16.3)	16 (30.2)
History of ovarian surgery**			
	**Yes**	33 (5.9)	24 (12.4)
	**No**	528 (94.1)	164 (87.2)
Kolmogorov-smirnov test as a benchmark for data abnormality. *Data presented as median range for the abnormally distributed data; **Data presented as mean for the normally distributed data. BMI: Body mass index; AMH: Anti-mullerian hormone			

**Table 3:** Association of age, BMI, endometriosis, and history of ovarian surgery with the poor-responder group


**Demographical and clinical characteristics**		**Good-responder group (n = 561)**	**Poor-responder group (n = 188)**	**p-value**	**OR; 95% CI**
Age (yr)*			
	**≥ 35 **	302 (53.8)	147 (78.2)	
	**< 35 **	259 (46.2)	41 (21.8)	< 0.001${}^{a}$	3.1 (2.1-4.5)
BMI (kg/m ${}^{2}$)**		23.63 (15.63-43.28)	23.37 (15.43-38.87)			0.174	
Endometriosis *					
	**Yes**	92 (16.4)	53 (28.2)	
	**No**	469 (83.6)	135 (71.8)	< 0.001${}^{a}$	2.0 (1.3-2.9)
Location of endometriosis*					
	**Ovarium**	67 (72.8)	30 (56.6)	
	**Extra ovarium**	10 (10.9)	7 (13.2)	
	**Both**	15 (16.3)	16 (30.2)	0.105	
History of ovarian surgery*					
	**Yes**	33 (5.9)	24 (12.4)		
	**No**	528 (94.1)	164 (87.2)	0.002${}^{a}$	2.3 (1.3-4.1)
*Chi-Square test; data presented as n (%). **Mann-Whitney U test; Data presented as Median range, BMI: Body mass index; CI: Confidence interval; a: Significant p-value					

**Table 4:** Multinomial regression analysis to predict Poseidon group


**Poseidon groups**	**BMI**		**History of surgery**		**Endometriosis**	
	**p-value**	**OR (95% CI)**	**p-value**	**OR (95% CI)**	**p-value**	**OR (95% CI)**
**Group 1**	0.45	—	0.808	—	0.013*	2.5 (1.2-5.2)
**Group 2**	0.304	—	0.007*	4.6 (1.5-13.9)	0.118	—
**Group 3**	0.024*	0.9 (0.8-0.99)	< 0.001*	10.7 (3.4-33.9)	0.208	—
**Group 4**	0.933	—	0.10	—	< 0.001*	2.7 (1.6-4.7)
BMI: Body mass index; CI: Confidence interval; *Significant with multinomial regression analysis used to classify Poseidon group based on the risk factor						

## 4. Discussion

In this study, an association between age ≥ 35 yr and poor responder was observed. A total of 78.2% participants aged ≥ 35 yr were at 3.1 (2.1-4.5) times higher risk for poor response than those aged < 35 yr. Several studies have used age ≥ 40 yr as a predictor of poor response. Whereas, some have used a cut-off value of 35 yr (Poseidon group) (14, 21). An increase in the BMI decreased the risk to be classified under Poseidon group 3. Halawaty and colleagues showed insignificant difference between obese and nonobese participants in terms of age, serum AMH level, serum FSH level, fasting blood glucose level, 2-h post-prandial blood glucose level, and AFC (22). Additionally, an increase in BMI was correlated to polycystic ovarium syndrome (PCOS) and higher AMH levels. Some study showed that most obese participants with PCOS had higher AMH level and metabolic disruption (23, 24). Therefore, an increase in BMI decreases the risk of poor response.

In terms of BMI, the poor-responder group had a higher BMI than the good-responder group. However, the result was not significantly different (p > 0.05). The association between obesity and ovarian reserve remains controversial. A study showed the negative effects of obesity on ovarian reverses (25). De Pergola *et al* explained that the follicle-stimulating hormone (FSH), luteinizing hormone (LH), inhibin B, and estradiol levels in women of reproductive age, who are either overweight or obese, were lower than those of women with normal weight in the early follicular phase due to the inhibitory effect of body mass on gonadotropin and estradiol production (26). However, several studies have shown that obesity is not associated with AFC. Moreover, lower AMH levels in obese women of reproductive age are attributed to physiological process and does not decrease the ovarian reserve (25-27).

In this study, endometriosis was significantly associated with poor responders (p < 0.001). The participants with endometriosis were at 2.0 (1.3-2.9) times higher risk to be a poor responder than those without. However, no association was observed between the location of endometriosis and poor response (p > 0.05). A small study reported that 34 women with endometriosis had lower AMH levels than those with infertility due to tubal factors (1.26 vs 2.02 ng/mL; p = 0.004) (28). Dokras and co-authors founded that inhibin B level was significantly lower in women with endometriosis than those without during gonadotropin stimulation (29). Hwu and colleagues and Uncu and co-workers also reported that the serum AMH level of women with endometriosis was significantly lower than the control group. However, they diagnosed endometriosis only by ultrasonography (30, 31). According to the Poseidon criteria, endometriosis was a significant predictor for Poseidon group 1. The participants with endometriosis were at 2.5 times higher risk to be classified under Poseidon groups 1 and 4 (95% CI: 1.5-5.2). Theoretically, endometriosis reduces ovarian reserve due to its inflammatory effects on the ovarian cortex based on serum AMH levels. The density of ovarian cortex in women with endometriosis decreases due to the formation of fibrotic tissues (32). A study showed that endometriosis cause a decrease in serum estradiol levels and production of LH-dependent progesterone (33).

Interestingly, a significant association was observed between a history of ovarian surgery and poor response (p < 0.05). Participants with a history of ovarian surgery were at 2.3 times higher risk to be poor responder (95% CI: 1.3-4.1). Lind and colleagues assessed the effect of cyst removal on ovarian reserve by measuring serum AMH levels (34). Another study also reported about a continuous decreased in serum AMH levels from 2.7 µg/L to 2.0 µg/L at a six-month follow-up after surgery and to 1.0 µg/L at two-year follow-up (34). A history of ovarian surgery was a significant predictor of classification under Poseidon groups 2 and 3. The participants with a history of ovarian surgery were at 4.6 times higher risk for poor response (95% CI: 1.5-13.9) than those without. The results of this study were similar to those of other studies showing a continuous decrease in serum AMH levels from 2.7 µg/L to 2.0 µg/L at a six-month follow-up after surgery and to 1.0 µg/L at a two-year follow-up (34).

## 5. Conclusion

Significant differences were observed in terms of age, endometriosis, and history of ovarian surgery in the poor-responder group. Endometriosis was a significant predictor of classification under Poseidon groups 1 and 4, and a history of ovarian surgery was a significant predictor of classification under Poseidon groups 2 and 3. Furthermore, BMI was a significant predictor of classification under Poseidon group 3. Age, BMI, endometriosis, and history of ovarian surgery were associated with ovarian reserve in the poor-responder group.

##  Conflict of Interest

The authors declare that they have no competing interests.
